# Specifics of Kandinsky–Clérambault syndrome with religious delusion of possession in schizophrenia

**DOI:** 10.1192/j.eurpsy.2022.1812

**Published:** 2022-09-01

**Authors:** E. Gedevani, G. Kopeiko, O. Borisova, U. Popovich

**Affiliations:** 1 FSBSI «Mental Health Research Centre», Researching Group Of Specific Forms Of Mental Disorders, Moscow, Russian Federation; 2 FSBSI Mental Health Research Center, Researching Group Of Specific Forms Of Mental Disorders, Moscow, Russian Federation; 3 Mental Health Research Center, Adolescent Psychiatry, Moscow, Russian Federation

**Keywords:** Kandinsky–Clérambault syndrome, schizophrénia, religious delusion, delusion of possession

## Abstract

**Introduction:**

Kandinsky–Clérambault syndrome with religious delusion of possession (KSRDP) in schizophrenia is insufficiently explored phenomenon. The syndrome characterized by significant severity of clinical state, high social risks and resistance to psychopharmacotherapy and requires the close attention.

**Objectives:**

To analyze psychopathological specifics of KSRDP and to identify the prognosis, dynamics of schizophrenia with KSRDP.

**Methods:**

Thirty four patients (18 women; 16 men; the average age 28 ± 9,5 years) with schizophrenia (F20.0, F20.01, F20.02 according to ICD-10) were examined by psychopathological, psychometrical and statistical methods

**Results:**

The specifics of the syndrome is delusional belief in possession by demonic or divine *‘spiritual being’,* invaded within the body. This possession is interpreted by patients as the totality of mind, body and soul control; and in several cases – as the appearance of a new identity. According to the “classical” Kandinsky–Clérambault syndrome, KSRDP accompanied by extensive psychic automatisms (ideational, cenestopathic, kinaesthetic), haptic and olfactory pseudo-hallucinations. Furthermore the specific hallucinations for KSRDP (*Hallucinationen der Gemeingefühlsempfindun* by von Krafft-Ebing, R.) are observed, which based on sensory-spatial imaginary sensations, with a clear localization in the field of a visceral sensitivity (as a material object with a certain shape, consistency, size, and weight).

**Conclusions:**

In contrast with “classical” paranoid syndrome of Kandinsky–Clérambault when negative effect is usually perceived by patients as external influence, KSRDP is characterized by delusional idea of ‘spiritual being’s invasion inside the body, mind and soul to control the whole human’s existence. Patients with KSRDP require specific treatment and management due to the religious content of delusion.

**Disclosure:**

No significant relationships.

